# Thrombotic Thrombocytopenic Purpura After Live Liver Donation: Villain or Scapegoat?

**DOI:** 10.7759/cureus.12890

**Published:** 2021-01-24

**Authors:** Madhumita Udayasankar, Ashwin Rammohan, AC Sathya, Akila Rajakumar, Mohamed Rela

**Affiliations:** 1 The Institute of Liver Disease & Transplantation, Dr Rela Institute and Medical Centre, Chennai, IND

**Keywords:** systemic lupus erythematosis, live liver donor, ethics, thrombotic thrombocytopenic purpura, outcomes

## Abstract

Living donor liver transplantation is a complex surgery, where the donor’s safety is of paramount importance. Despite all precautions, donor morbidity may be inevitable, and long-term follow-up data attest to this fact. However, being a “past donor” all ailments are intuitively attributed to the donation process, which may not always be the case. We present the case of a 47-year-old lady, who developed thrombotic thrombocytopenic purpura secondary to systemic lupus erythematosus 18 months following her liver donation, when she detected to be anti-nuclear antibody (ANA) positive. She developed neurological signs and was managed successfully with therapeutic plasma exchange and steroids. She was discharged home on immunosuppression and remains well on follow-up. We present the medical and social issues that were addressed in the case and highlight the need for a more stringent follow-up protocol in those who are ANA positive. This would also help detect morbidities that may be unrelated to the donation process.

## Introduction

Apart from its complex ethical issues, living donor liver transplantation (LDLT) remains a technically demanding procedure. In these surgical procedures, donor safety is of paramount importance, as are recipient outcomes. Donor evaluation is aimed at revealing conditions that could increase the risk of peri-operative complications in a healthy person. This systematic and algorithmic evaluation process excludes unsuitable prospective donors at an early stage, while allowing for suitable candidates to proceed towards donation. This basic concept of minimizing donor risk remains essential to any living donor program. However, despite all precautions, a small risk of complications to the donor remains a reality [1,2].

There is sizable literature documenting the likelihood of medical and psycho-social decrements after live liver donation. A meta-analysis looking at long-term physical health of live donors (LDs) showed that well over half of all donors (in certain series up to 75%) reported ongoing donation-related health problems, primarily related to gastrointestinal issues, incisional and abdominal pain, and hernia [3]. Notwithstanding this sobering statistic, medical issues unrelated to the donation may also crop up. Being a past donor, each physical ailment intuitively gets linked to the donation process, clouding the medical, psycho-social and emotional aspects of their treatment.

We present one such case of an LD who developed thrombotic thrombocytopenic purpura (TTP) secondary to systemic lupus erythematosus (SLE), and in turn discuss the various issues which were addressed in solving the conundrum.

## Case presentation

A 47-year-old lady presented with a four-day history of lethargy, vomiting and myalgia. She also gave a history of fever that subsided spontaneously one week prior to her admission. On further elicitation, she complained of alopecia and weight loss over the previous two months. She did not have any history of joint pain, skin rash, altered sensorium or seizures.

Eighteen months prior to this episode, she had donated the left-lateral segment of her liver to her grandson who underwent a living donor liver transplant (LDLT) for congenital hepatic fibrosis. Her donor evaluation was unremarkable apart from a positivity of anti-nuclear antibody (ANA) (1:1000 titre). Further evaluation of her autoimmune markers including those for autoimmune hepatitis (ASMA, AMA, Ab to LKM1) was negative. A pre-operative liver biopsy was performed, which was also normal. The donor operation and her post-operative period was uneventful and her routine outpatient donor follow-up (three monthly for the first six months, then annually) since the operation had been unremarkable.

Immediately prior to the current admission, she was evaluated at two local health facilities, both of which were wary of her “past-donor” status and referred her on. At admission, her blood tests were suggestive of severe bicytopenia with a haemoglobin of 4g/dL (N=12-15g/dL) and platelet count of 10,000/mm^3^ (N=150,000-450,00/mm^3^) along with acute kidney injury with a serum creatinine of 2.5mg/dL (N=0.6-1.2mg/dL). Abdominal imaging showed a well regenerated liver with normal vasculature and her liver function tests were normal apart from mildly elevated indirect bilirubin levels of 1.5mg/dL (N=03-1.2mg/dL). In the ward, she had an episode of transient loss of consciousness without focal neurological deficit. MRI brain revealed an acute left capsuloganglionic infarct. Further blood tests in the form of peripheral smear (abundance of schistocytes), elevated LDH, high reticulocyte count, low haptoglobulin and indirect hyperbilirubinemia were suggestive of microangiopathic haemolytic anemia. Autoantibody screen revealed anti-dsDNA and extractable nuclear antigen panel (anti-SnRNP, anti-SM and anti-Jo 1) antibodies to be positive.

Putting together her constellation of symptoms and signs, a diagnosis of TTP secondary to SLE was made. Her thrombocytopenia remained refractory despite multiple transfusions. Her PLASMIC score of 6 (a scoring system to stratify the risk of TTP based on a composite of platelet count, hemolysis, the presence of cancer, history of solid organ or stem-cell transplant, mean corpuscular volume, international normalised ratio (INR) and creatinine) put her in the high-risk category. Following a multi-disciplinary team discussion, as per standard risk-stratified guidelines for the management of TTP, she was started on emergency therapeutic plasma exchange along with high dose steroids [4]. Hydroxycholorquine (HCQ) and IV cyclophosomide were also added to her list of medications. She became symptomatically better and her platelet counts and LDH levels improved with four cycles of plasma exchange. Her serum creatinine also normalized. She was discharged home on HCQ and corticosteroids and remains well on follow-up. The recipient is asymptomatic with a stable graft function.

## Discussion

The finite risk of complications and/or death in an otherwise healthy live donor is the single biggest detriment to performing LDLTs. There is also a growing international consensus that the long-term impact of LD demands greater attention in both research and clinical arenas. LD evaluation involves a multi-step protocol that includes exhaustive medical and psychological evaluations of the donor, as well as a precise anatomical study of the liver. LDLT is a directed organ donation and a clear judgment is needed to balance the donor risk versus the recipient benefit with the aim to satisfy the tenet of “double equipoise” [2]. One of the debatable aspects of the evaluation is when the prospective donor has a co-morbidity or a deranged blood test.

To the best of our knowledge, this is the first reported case of SLE with secondary TTP occurring after live liver donation. During the donor evaluation process, our patient was detected to be ANA positive. Despite this, the decision to proceed with her donation was based on the fact that her other autoimmune markers and liver biopsy were normal. Various series have shown 4%-26% of otherwise normal individuals to be ANA positive. On long-term follow-up, 0.3% to 6% of those who are ANA positive go on to develop symptomatic disease [5]. Unfortunately, there are no set guidelines for the evaluation or management of this incidentally ANA positive cohort. SLE is the disease most intimately linked to ANA positivity and women in the age group of 15 to 45 years have the highest propensity for developing it. In retrospect, we are unlikely to have changed our decision of offering a liver donation to this patient.

TTP, per se whether idiopathic or secondary, has a high mortality of around 90% in untreated individuals and therapeutic plasma exchange along with immunosuppression remain the main modalities of treatment. Inciting factors of TTP include infections, pregnancy, surgery and hematopoietic stem cell transplant [6]. There are multiple case reports of TTP being triggered after surgery. This, however, is unlikely in our patient due to the long lag period between the liver donation and the TTP. Since ours is the first such report, we had no previous similar reports to base our treatment. Nevertheless, notwithstanding her "past-donor" status, the management of TTP did not differ from those in published guidelines [4]. 

Another interesting consideration is that any physical ailment in a past donor is automatically attributed to the donation process. Many healthcare units are wary of treating these patients. Despite the well-published long-term morbidity to these donors, it is imperative to understand that not all illnesses that occur during the lifetime of a past donor can be attributed to the donor operation. Our patient highlights this tenet, wherein she was referred to our unit from two different regional centers who were unsure of evaluating her medical condition as she was a liver donor. Failure or delay in diagnosis could have been catastrophic to our patient. In cases where health insurance is involved, the “past-donor" epithet could have a major financial impact on the patient’s future medical care.

LDLT programs world over have donor follow-up schedules to detect physical and psycho-social issues that may afflict this cohort. Despite the patient’s adherence to our follow-up protocol, we were unable to pre-emptively detect the evolution of SLE in our patient. Reflecting on this case, we feel it might be prudent to have a modified follow-up protocol for a subgroup of donors like those who are ANA positive.

## Conclusions

We present a case of TTP secondary to SLE in a liver donor who was previously ANA positive. This case posed both medical and social issues that required addressing. Transplant programs often feel responsible for illnesses that arise in donors both in the short and long term even though they may not entirely be related to the donation process. Extensive workup during the donation process often throw up abnormalities that are incidental and do not represent a contraindication for the donor operation. In this instance, some abnormality was indeed detected in the donor, which did not amount to contraindication for the donation process. However, a more stringent follow-up may have identified early signs of SLE in this patient. This case illustrates the need for appropriate referral and focused follow-up in the long term when such abnormalities are detected.
